# Publisher Correction: Microtubule disassembly by caspases is an important rate-limiting step of cell extrusion

**DOI:** 10.1038/s41467-022-32068-8

**Published:** 2022-07-27

**Authors:** Alexis Villars, Alexis Matamoro-Vidal, Florence Levillayer, Romain Levayer

**Affiliations:** 1 Department of Developmental and Stem Cell Biology, Institut Pasteur, Université de Paris Cité, CNRS UMR 3738, 25 rue du Dr. Roux, 75015 Paris, France; 2 Sorbonne Université, Collège Doctoral, F75005 Paris, France

**Keywords:** Apoptosis, Microtubules, Morphogenesis

Correction to: *Nature Communications* 10.1038/s41467-022-31266-8, published online 25 June 2022

The original version of the Supplementary Information associated with this Article included an incorrect Description of Additional Supplementary Files, which was missing all the movie legends. The HTML has been updated to include a corrected version of the Description of Additional Supplementary Files.

## Supplementary information

Updated Supplementary Information

