# Supplementary material for: Microtubule disassembly by caspases is an important rate-limiting step of cell extrusion
Source: Nat Commun. 2022 Jun 25;13:3632. doi: 10.1038/s41467-022-31266-8 (PMC9233712; doi:10.1038/s41467-022-31266-8)
Supplement: Supplementary file 1 — Supplementary Information [file 41467_2022_31266_MOESM1_ESM.pdf]

# Supplementary figures

## Microtubule disassembly by caspases is an important rate-limiting step of cell extrusion

Alexis Villars[1,2], Alexis Matamoro-Vidal[1], Florence Levillayer[1] and Romain Levayer[1*]

1. Department of Developmental and Stem Cell Biology, Institut Pasteur, Université de Paris Cité, CNRS UMR 3738, 25 rue du Dr. Roux, 75015 Paris, France

2. Sorbonne Université, Collège Doctoral, F75005 Paris, France

* Correspondance to: romain.levayer@pasteur.fr

# Supplementary figure 1

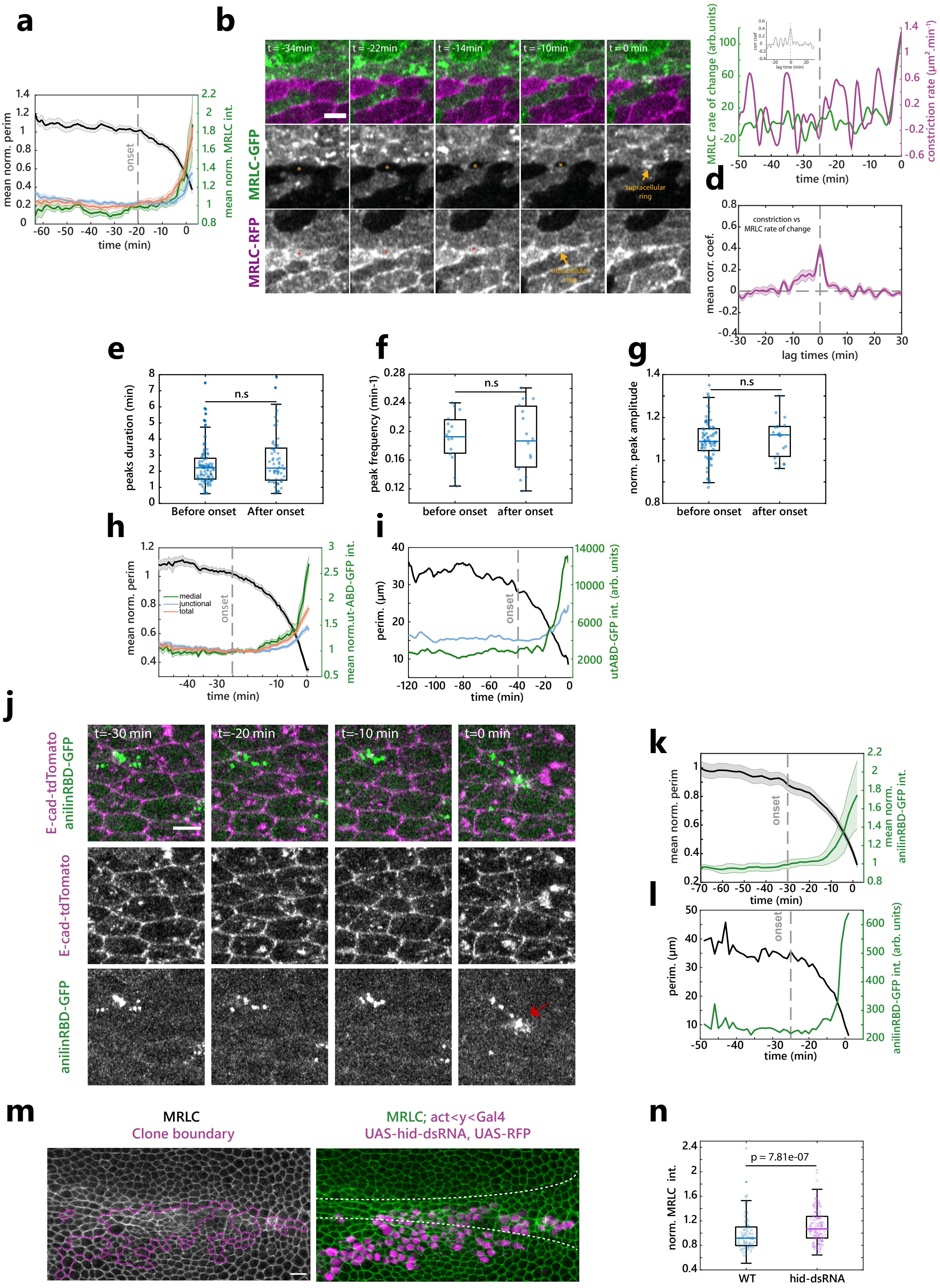

**Supplementary figure 1: Myosin concentration and dynamics do not change at the onset of extrusion**

**a** Normalised averaged MRLC intensity (sqh-GFP) in different pools (medial, green, junctional, blue, total, orange) and perimeter (black) in midline extruding cells. Dotted line, onset of extrusion. Light colour areas, S.E.M.. n=15 cells. **b** Actomyosin ring marked with mitotic clones expressing either MRLC-GFP, MRLC-RFP or both. Top, overlay, middle, MRLC-GFP, bottom, MRLC-RFP. Orange and red stars, same extruding cell. Arrows, rings formation. Scale bar, 5μm. **c** Representative curve of MRLC-GFP intensity rate of change (green) and perimeter contraction rate (purple) in an extruding cell. Inset, normalised cross-correlation of these curves. t0, onset of extrusion (dotted line). N= 2, n>16 cells. **d** Average normalised cross-correlation of MRLC rate of change and perimeter contraction rate. n=15 cells. Light area, S.E.M.. **e,f,g** Boxplots showing MRLC intensity peaks duration (half-peak width, **e,** n=106 & 61 pulses), frequency (**f,** n=15 cells) and normalised amplitude (**g**, n>70 and 20 peaks, before or up to 10 min after extrusion onset), before and after extrusion onset. n.s.: non-significant. For each boxplot, central line, median value, bottom and top of the box 25th and 75th percentiles. Whiskers, most extreme points not considered outliers. Outliers shown in red. **h** Averaged normalised F-actin intensity (utABD-GFP) in different pools (medial, green, junctional, blue, total, orange) and perimeter (black). Light colour areas, S.E.M.. Grey dotted line, onset of extrusion. n=37 cells. **i** Representative curve of junctional (blue) and medial (green) utABD-GFP intensity and perimeter (black) of an extruding cell. **j** Anillin Rho-Binding-domain-GFP during cell extrusion (green, bottom row) and E-cad-tdTomato (magenta, middle). Red arrow, late Rho accumulation. Scale bar, 5μm. **k** Averaged normalised AnilinRBD-GFP apical signal (green) and perimeter (black) during cell extrusion. Grey dotted line, extrusion onset. Light colour areas, S.E.M.. N=2 pupae, n=31 cells. **l** Single representative curve of AnnilinRBD-GFP intensity (green) and perimeter (black) of an extruding cell. **m** MRLC-GFP (green, left) with hid-dsRNA clones (magenta, magenta lines, clone borders). White doted lines, midline. Scalebar, 10μm. **n** Boxplot of MRLC-GFP junctional intensity (one dot per cell) outside or inside hid-dsRNA clones. p-value = 7.8129e-07. N=2 pupae, n =779 WT and 155 clonal cells (only a subset of points shown). All statistical tests: two-sided t-tests. Source data are provided in the source data file.

# Supplementary figure 2

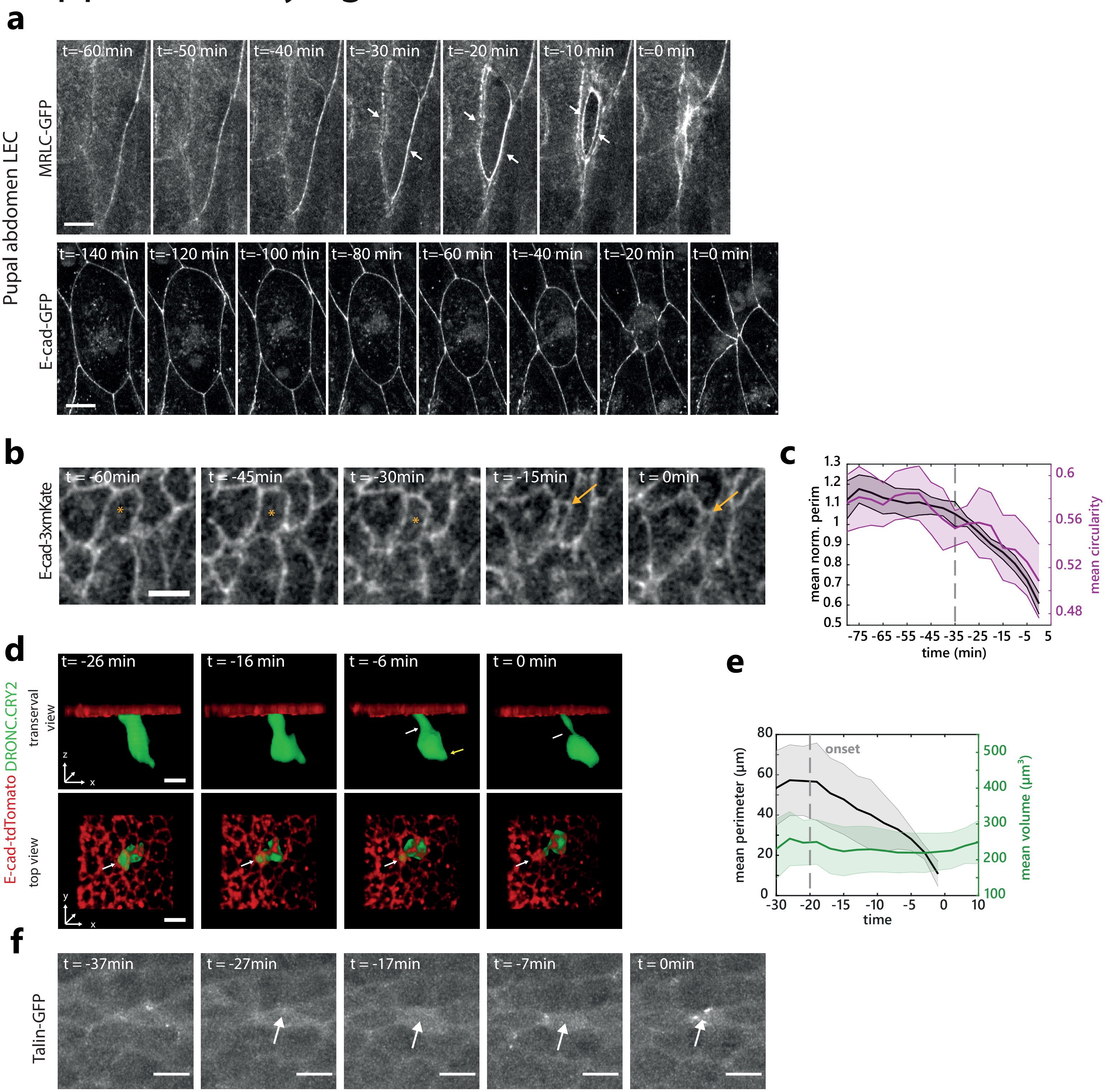

**Supplementary figure 2: Cell extrusion in the notum is not initiated by an actomyosin ring, nor volume reduction or a modulation of ECM binding**

**a** Snapshots of extruding larval epidermal cells (LEC) from the pupal abdomen. Top shows MRLC (sqh-GFP). N=2, n=27 cells. White arrows, supracellular and intracellular myosin ring. N=1, n=10 cells. Bottom, evolution of cell perimeter visualised with E-cad-GFP which shows cell rounding. Scalebar, 10µm. **b** Snapshots of E-cad-3xmKate during the extrusion of a cell in ROCK-dsRNA (driven by pnr-Gal4). Stars point at the extruding cell. Arrows point at the final stage of cell extrusion where the cell decrease its circularity. Scale bar, 5µm. N=2, n>10 cells. **c** Averaged and normalised perimeter (black) and circularity (purple) during cell extrusion in ROCK-dsRNA context. Dotted line, inflexion point. Shadowed area are S.E.M. n=10 cells. **d** Snapshots of a single extruding cell upon optoDronc activation with 3D rendering (using cytoplasmic GFP signal). Green, segmented cell volume, red, cell contour visualised with E-cad-tdTomato. Top, transversal view, bottom, view from the apical side. White arrows point at apical reduction of volume while yellow arrow shows the basal increase of volume. Scale bars, 5µm. N=2, n=13 cells. **e** Averaged cell volume (green) and perimeter (black) during cell extrusion. Light colour areas are S.E.M.. n=6 cells. **f** Snapshots of Talin-GFP during cell extrusion. t0 is termination of extrusion. White arrows show an extruding cell. Scale bars, 5µm. Source data are provided in the source data file. N=1, n=15 cells.

# Supplementary figure 3

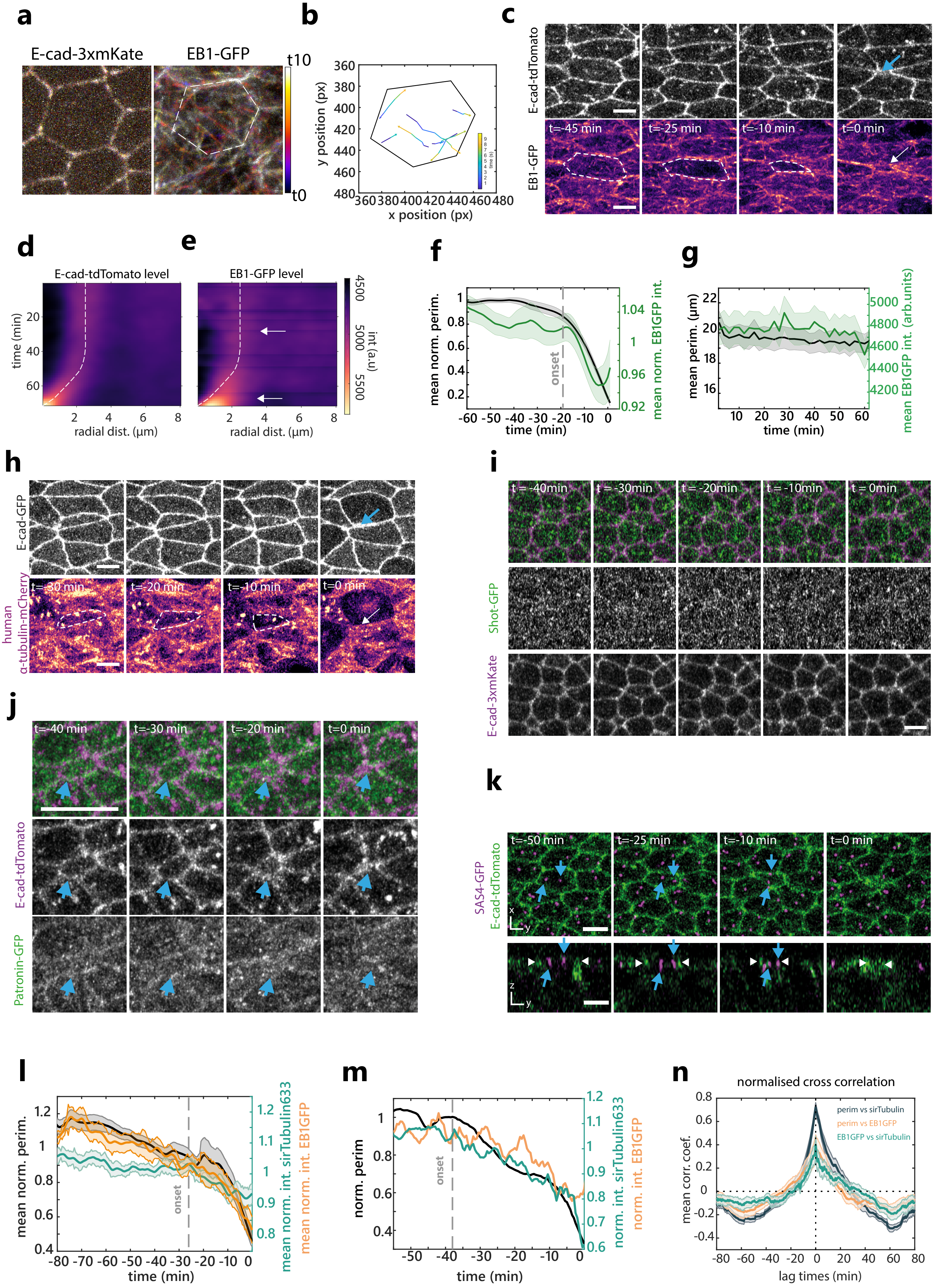

**Supplementary figure 3: Visualisation of microtubule depletion using different markers.**

**a** Time projection (colour-coded) of E-cad-3xmKate (left) and EB1-GFP (right) over 10s. Dotted white line, cell contour. N=2, n>50 cells. **b** Tracking of EB1 comets over 10s. **c** E-cad-tdTomato (top) and EB1-GFP (bottom, pseudo-colour) during cell extrusion in the midline. Blue arrow, end of extrusion, white arrow, late EB1 accumulation in the neigbours, cell contour, white dotted lines. Scale bar, 5µm. N=2, n=60 cells. **d,e** Radial averaged kymograph (see **Figure 3g-i**) of E-cad-tdTomato (left) and EB1-GFP (right) in pseudo-colour, time on y-axis going downward, x-axis, radial distance from cell center. White dotted lines, average cell contour (detected with the maximum of E-cad signal). Top white arrow, onset of EB1 depletion in the extruding cells, bottom arrow, late EB1-GFP accumulation in the neighbours. n=60 cells. **f,g** Averaged normalised EB1-GFP signal (green) and perimeter (black) in extruding cells (**f**, n=22) and non-extruding cells (**g**, n=27). Grey dotted line, onset of extrusion. Light colour areas, S.E.M.. **h** E-cad-GFP (top) and human αTubulin-mCherry (bottom, pseudo-colour) during cell extrusion. Red and white arrows, end of extrusion. White doted lines, extruding cell contours. Representative of 15 cells. Scale bar, 5µm. **i** Shot-GFP (green, middle panel) and E-cad-tdTomato (magenta, bottom panel) in an extruding cell. t0, termination of extrusion. Representative of 20 cells. Scale bar, 5µm. **j** Patronin-GFP (green, bottom) and E-cad-tdTomato (magenta, middle) in an extruding cell (blue arrows). t0, termination of cell extrusion. Representative of 20 cells. Scale bar, 10µm. **k** Centrosome position during cell extrusion visualised by SAS4-GFP (magenta) with E-cad-tdTomato (green) top and lateral view. t0, termination of cell extrusion. Blue arrows, centrosome positions. White arrows, junctions of the extruding cell. Representative of 15 cells. Scale bar, 5µm. **l** Averaged and normalised perimeter (black), sirTubulin633 intensity (green), EB1-GFP intensity (orange) during extrusion. Grey dotted line, onset of extrusion. Light colour areas, S.E.M.. n=19 cells. **m** Perimeter (black), sirTubulin633 intensity (green) and EB1-GFP intensity (Orange) of an extruding cell. Grey dotted line, onset of extrusion. **n** Averaged and normalised cross-correlation of the perimeter vs sirTubulin633 (black) or vs EB1-GFP (orange) or EB1-GFP vs sirTubulin633 (green). Light colour areas, S.E.M.. Maximum of correlation at t=0min for each curve. n=19 cells. Source data are provided in the source data file.

# Supplementary figure 4

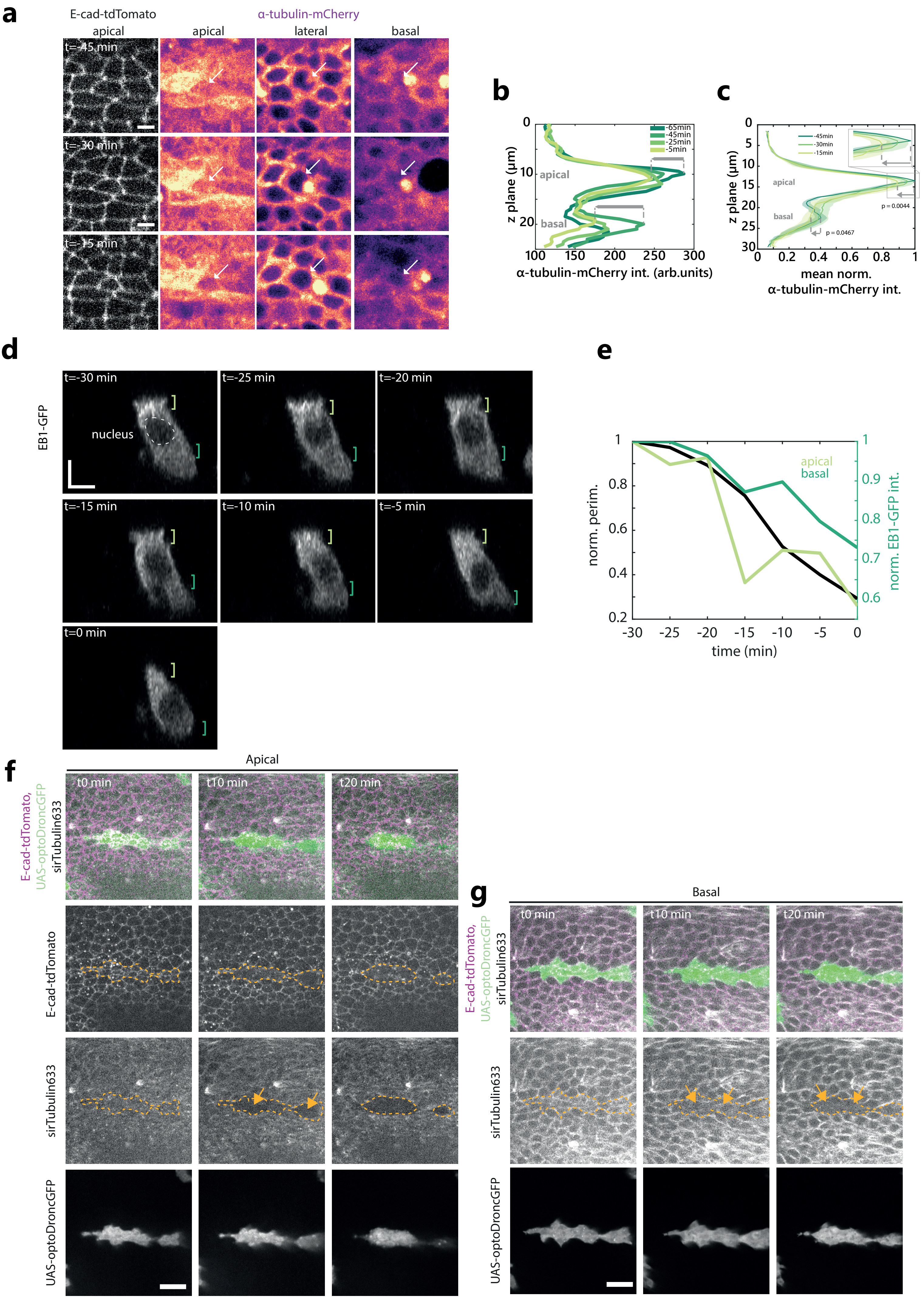

**Supplementary figure 4: MT depletion occurs throughout the cell.**

**a** α-tubulin-mCherry in different z-planes during cell extrusion (apical, lateral and basal, time from top to bottom, t0, termination of extrusion). Left panels, E-cad-tdTomato in the junction plane. White arrows, extruding cell in every plane. Scale bars, 5µm. N=2, n=11 cells. **b,c** Quantification of apico-basal αtubulin-mCherry signal during extrusion (y-axis, z-plane, x-axis signal, different colour for each time point, t0 extrusion termination). **b** Single representative quantification for the cells shown in **a**. Grey lines, reduction of signal over time apically and basally. **c** Averaged and normalised quantification of αtubulin-mCherry apico-basal signal during cell extrusion. Axis are the same as in **b**. The inset highlights apical depletion. Grey arrows, signal reduction in the apical and basal plane. n=11 cells. p-values are pairwise and one-sided t-test. Light colour areas, S.E.M.. **d** Side views of a single-cell clone expressing UAS-EB1-GFP during cell extrusion. Light green bracket, apical plane, darker green, basal plane bellow the nucleus. Nucleus boundary highlighted with the white dotted line. N=2, n=24 cells. **e** Perimeter (black), and EB1-GFP normalised intensity apically (light green) or basally (darker green) of the single extruding cell example shown in **d**. Representative of 10 cells. **f,g** Snapshots of the apical and basal depletion of sirTubulin633 (grey, middle panel) of a clone expressing UAS-optoDronc (green, bottom panel) after blue light exposure (t0 to t20min). N=2, n=12 clones. **f** Snapshots of the apical plane of that clone visualised using Ecad-tdTomato (magenta, top panel). Orange dotted line, border of the clone. Orange arrows, extruding/dying cells where tubulin signal is depleted. **g** Snapshots of a basal plane of that clone. Orange dotted line, border of the clone. Orange arrows, extruding/dying cells where tubulin signal is depleted (the cytoplasm of the cells can be seen with the GFP channel). Scale bars are 10µm. Source data are provided in the source data file.

# Supplementary figure 5

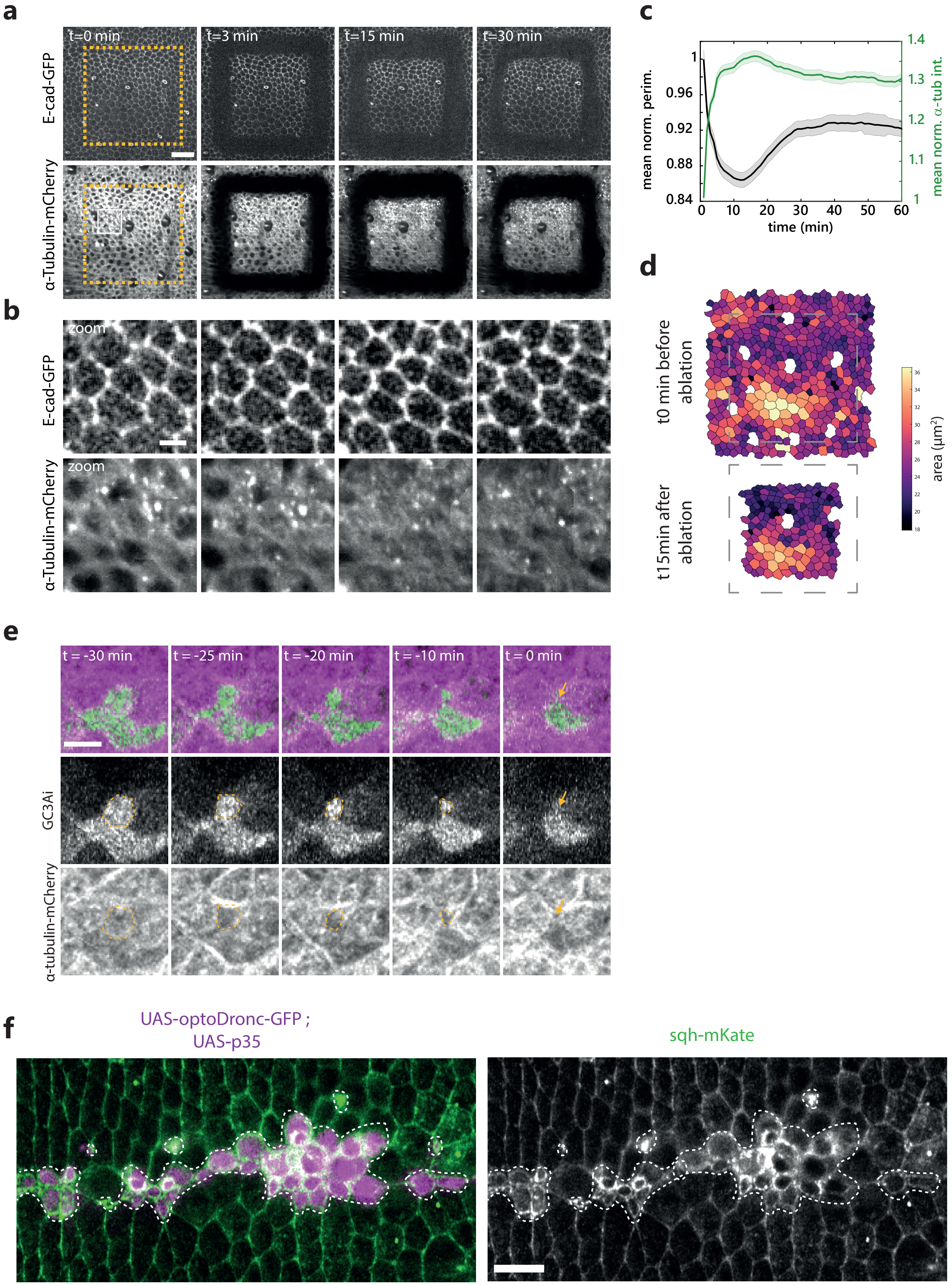

**Supplementary figure 5: The depletion of microtubules is effector caspases dependent**

**a,b** Snapshots of a laser ablated pupal notum. **a** Snapshots of the full ablated region (orange dotted square) showing α-tubulin-mCherry (bottom) and E-cad-GFP (top). t0 is the last time point before ablation (note the relaxation at t=15min). Scale bar, 25µm. N=3 pupae. **b** Snapshots of a zoom in a sub-region in the ablated region (White Square in **a**) showing α-tubulin-mCherry (bottom) and E-cad-GFP (top). Scale bar, 5µm. **c** Averaged and normalised α-tubulin-mCherry signal (green) and cell perimeter (black) in the relaxed square region following ablation. Note the transient increase of Tubulin signal during the transient reduction of cell perimeter. Light colour areas are S.E.M.. N=3 pupae, n=123 cells. **d** Snapshots showing the segmentation of the cells in the ablated region and their cell area before ablation (t0min) or at maximal relaxation (t15min). Grey dotted line shows the ablation region. **e** Snapshots of caspase activation using GC3Ai (green, middle) before microtubule depletion visualised with α-tubulin-mCherry (magenta, bottom) during cell extrusion. Caspase activation (t=-30min) precedes the beginning of MT depletion (t=-25min). Orange dotted lines, contour of the extruding cell. Orange arrow, termination of extrusion. Representative of 20 cells and 2 movies, scale bar, 5µm. **f** Snapshot of MRLC accumulation (sqh-mKate, green and bottom) in clone expressing UAS-OptoDronc-GFP and UAS-p35 (magenta, contours shown with white dotted lines) after 3hours of blue light exposure. Representative of >10 clones. Scale bar is 10 µm. N=18 pupae. Source data are provided in the source data file.

# Supplementary figure 6

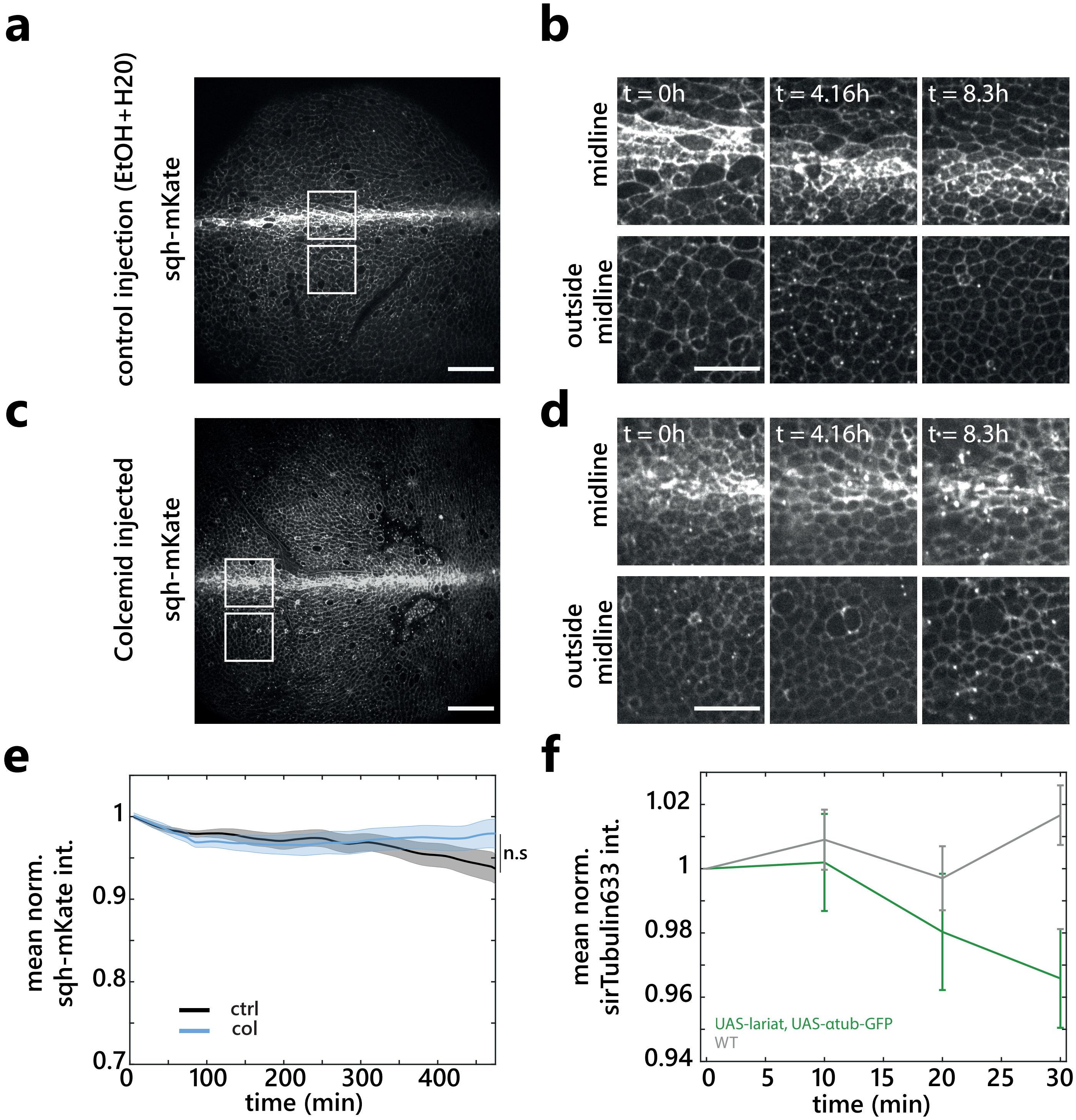

**Supplementary figure 6: Evolution of MRLC levels upon colcemid injection and LARIAT control**

**a-d** Snapshots of local z-projections of live pupal nota of Sqh-mKate (MRCL) levels following mock injection (EtOH+H20) (**a,b**) or colcemid injection (**c,d**) with the same acquisition parameters and contrasts. **a,c** Full nota view right after injection, Scale bars, 50µm. White boxes highlight the midline (top) and out-of-midline (bottom) regions shown in **b** and **d**. **b,d** Snapshots over time after injection. Scale bars, 25µm. **e** Normalised and averaged Sqh-mKate signal from total fluorescence of 3 regions per pupae after injection in colcemid (blue) or control (black) injections. Light colour areas are S.E.M.. N=2 pupa for control and 3 pupa for colcemid. **f** Normalised and averaged sir-tubulin633 levels in cells expressing UAS-Lariat, UAS-α-tubulin-GFP (green) or controls (grey) after blue light exposure. N = 2 nota, n = 20 wt cells, n = 15 clonal cells (UAS-Lariat, UAS-α-tubulin-GFP, green). Error bars are S.E.M.. Source data are provided in the source data file.

# Supplementary figure 7

**a**

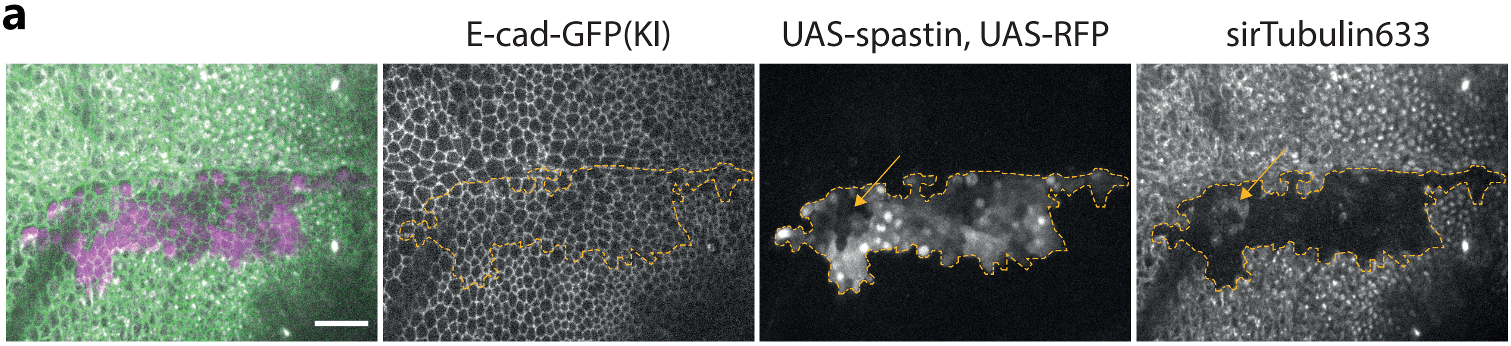


**b**

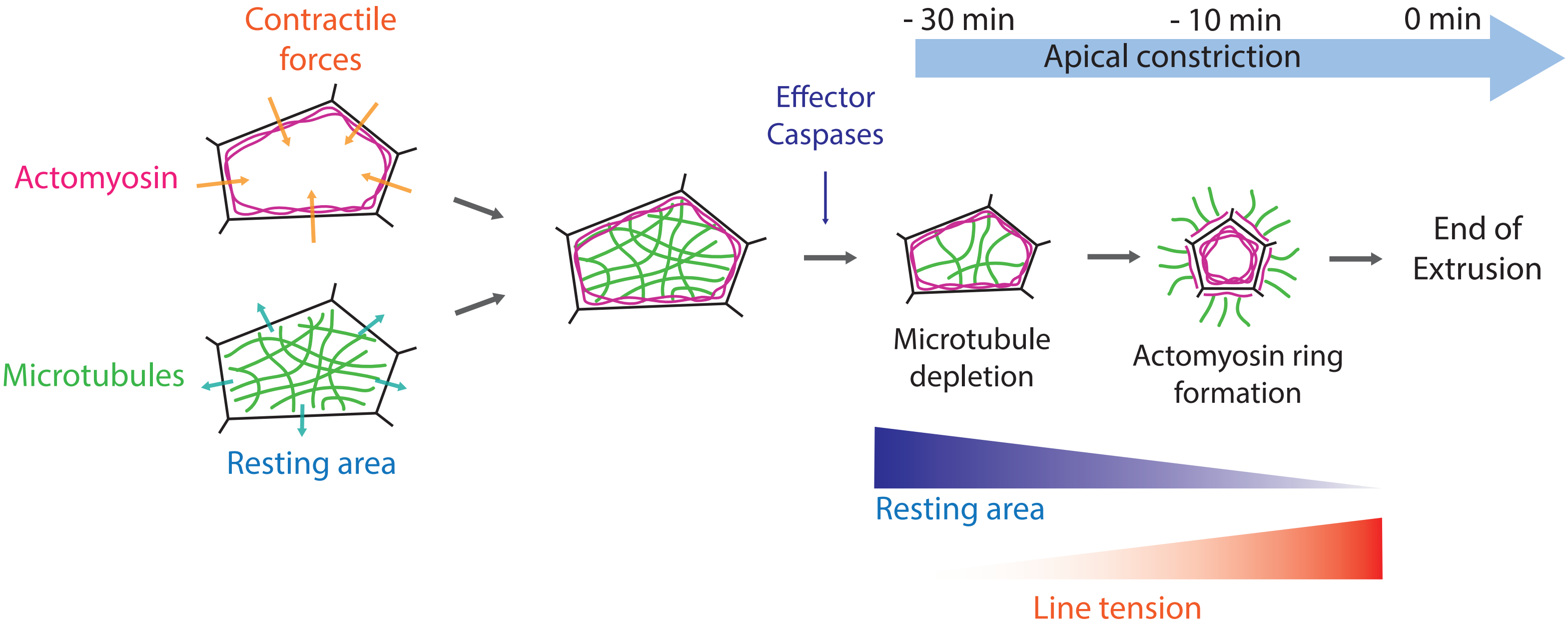

**Supplementary figure 7: Spastin depletion of Microtubules and working model**

**a** Snapshots showing sirTubulin633 depletion (grey, right) in clones expressing UAS-Spastin, UAS-RFP (magenta, middle) with tub-Gal80ts in the notum after 6 hours at 29°C (permissive temperature). Cell contour is visualised using E-cad(KI)-GFP (green, left). Orange dotted lines highlight the border of the clone. Orange arrows point at cell expressing less UAS-Spastin, UAS-RFP where the depletion of SirTubulin633 is weaker. Scale bar, 25µm. N=3 pupae. **b** Schematic of the working model. Cell-cell junctions are in black. Actomyosin is in light red and microtubules (MTs) are represented as green lines. Contractile forces are regulated by the junctional actomyosin network. MTs may regulate directly or indirectly the resting area of the cell. At equilibrium the two components are balanced. Upon effector caspases activation, MTs are progressively depleted, hence reducing the resting area of the cell and promoting apical constriction without cell rounding. Later on, actomyosin accumulates in a contractile ring which terminates cell extrusion through an increase of line tension and correlating with an accumulation of MTs in the neighbours. Source data are provided in the source data file.

# Supplementary figure 8

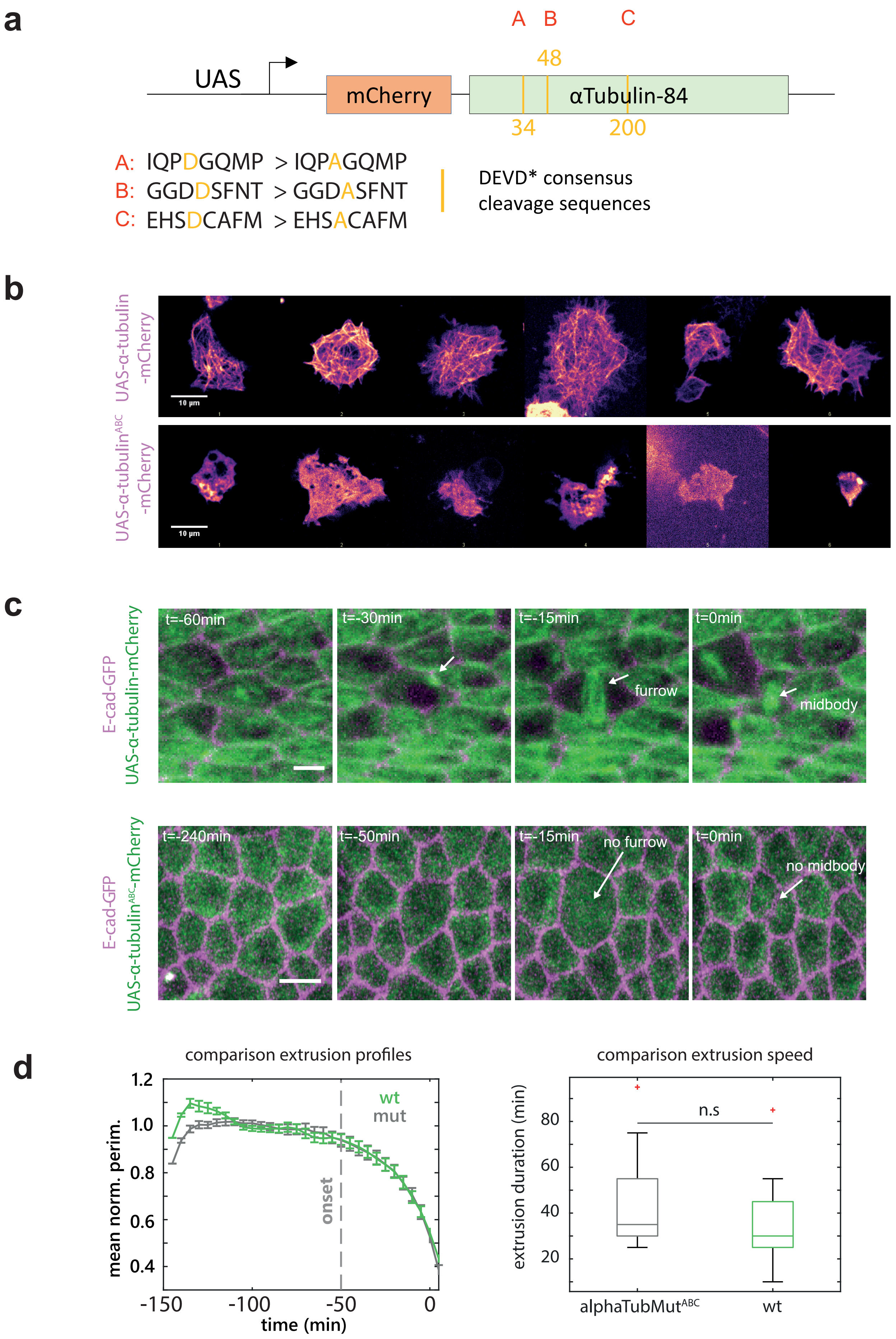

**Supplementary figure 8: Mutation of αTubulin caspase cleavage sites prevents proper integration in MTs**

**a** Schematic representation of the α-Tubulin-mCherry construct. A,B,C shows the different caspase-cleavage sites at 34, 48 and 200 amino acids respectively. Yellow bars show mutation sites. The sequence of the cleavage site and the introduced mutations are shown below. n=6 cells. **b** Snapshots of different representative S2 cells transfected with the WT αTubulin-mCherry construction (top) or the mutated form (labelled α-Tubulin$^{ABC}$-mCherry, bottom) driven by the UAS promoter. White arrows point at MTs in the WT form. These are absent from the cells expressing the mutant α-Tubulin$^{ABC}$-mCherry (mostly cytoplasmic signal). n=6 cells. **c** Comparison of the α-Tubulin-mCherry and α-Tubulin$^{ABC}$-mCherry localisation in the *Drosophila* pupal notum (UAS promoter, pnr-gal4 driver). Top row shows αTubulin-mCherry WT (green) with E-cad-GFP (magenta). White arrows point at one example of division furrow and the persisting midbody after cytokinesis. Bottom row shows mutant α-Tubulin$^{ABC}$-mCherry. White arrows point at the absence of labelling at the division furrow or the midbody. Scale bars are 5µm. **d** Left panel, Averaged and normalised apical perimeter of extruding cells expressing either WT UAS-α-Tubulin-mCherry (green) or mutant UAS-α-Tubulin$^{ABC}$-mCherry (grey). Error bars are s.e.m.. Grey dotted line shows the onset of extrusion. Cells are aligned by the end of extrusion. n= 30 cells for both conditions. Right panel shows box blot of extrusion duration (between initiation and full apical closure) of cell expressing either UAS-α-Tubulin-mCherry (green) or UAS-α-Tubulin$^{ABC}$-mCherry (grey). The difference is not significant (n.s.). n= 30 cells for both conditions. For each boxplot, the line in the middle represents the median value, the bottom of the box 25th percentiles and the top of the box is the 75th percentiles. The whiskers extend to the most extreme data points not considered outliers. If any, the outliers are plotted in a red marker. Two-sided t-test was performed. Source data are provided in the source data file.
