# Supplementary material for: Microtubule disassembly by caspases is an important rate-limiting step of cell extrusion
Source: Nat Commun. 2022 Jun 25;13:3632. doi: 10.1038/s41467-022-31266-8 (PMC9233712; doi:10.1038/s41467-022-31266-8)
Supplement: Supplementary file 3 — Description of additional Supplementary File [file 41467_2022_31266_MOESM3_ESM.pdf]

Inventory of supporting information

Microtubule disassembly by caspases is an important rate-limiting step of cell extrusion

Alexis Villars[1,2], Alexis Matamoro-Vidal[1], Florence Levillayer[1] and Romain Levayer[1*]

1. Department of Developmental and Stem Cell Biology, Institut Pasteur, Université de Paris Cité, CNRS UMR 3738, 25 rue du Dr. Roux, 75015 Paris, France

2. Sorbonne Université, Collège Doctoral, F75005 Paris, France

* Correspondance to: romain.levayer@pasteur.fr

The Supplementary files contain:

- The fused Supplementary figures including 8 Supplementary figures and their legends. We list them below:

Supplementary figure 1: Myosin concentration and dynamics do not change at the onset of extrusion

Supplementary figure 2: Cell extrusion in the notum is not initiated by an actomyosin ring, nor volume reduction or a modulation of ECM binding

Supplementary figure 3: Visualisation of microtubule depletion using different markers

Supplementary figure 4: MT depletion occurs throughout the cell.

Supplementary figure 5: The depletion of microtubules is effector caspases dependent

Supplementary figure 6: Evolution of MRLC levels upon colcemid injection and LARIAT control

Supplementary figure 7: Spastin depletion of Microtubules and working model

Supplementary figure 8: Mutation of αTubulin caspase cleavage sites prevents proper integration in MTs

- 21 Supplementary movies, whose titles and legends are given below:

Supplementary Movie 1: E-cad evolution during cell extrusion

Local projection of E-cad-GFP in an extruding cell from the midline region of the pupal notum. Scale bar is 5µm.

Supplementary Movie 2: MRLC evolution during cell extrusion

Local projection of sqh-GFP (MRLC) in an extruding cell from the midline region of the pupal notum. Scale bar is 5µm.

Supplementary Movie 3: Actin evolution during cell extrusion

Local projection of utABD-GFP in an extruding cell from the midline region of the pupal notum. Scale bar is 5μm.

Supplementary Movie 4: Localisation of Rho during cell extrusion

Local projection of aniRBD-GFP (Rho localisation, left, green) and E-cad-tdTomato (right, magenta) in an extruding cell from the midline region of the pupal notum. Scale bar is 5μm.

Supplementary Movie 5: Vertex model based simulations of early steps of cell extrusion

Left panel: control simulation. The tracked cells (blue) have parameters values identical to all the other cells. **Middle panel:** purse-string driven extrusion. At t = 20 sts, the tracked cells (blue) were forced to initiate extrusion by increasing at each iteration their contractility parameter ($\tilde{\Gamma}$) with a fixed rate ($\tilde{\Gamma}_{t+1} = \tilde{\Gamma}_t + 7.5.10^{-7}. \tilde{\Gamma}_t$). **Right panel:** At t = 20 sts, the tracked cells were forced to initiate extrusion by decreasing after each iteration their resting area ($A_\alpha{}^{(0)}$) with a fixed rate ($A_\alpha{}^{(0)}{}_{t+1} = A_\alpha{}^{(0)}{}_t - 3.5.10^{-4}. A_\alpha{}^{(0)}{}_t$).

Supplementary Movie 6: Evolution of cell volume during extrusion

3D rendering (top view left, lateral view, right) of an extruding cell upon activation of caspases by optoDronc. E-cad-tdTomato in red and cytoplasmic GFP in green. Scale bar is 5μm.

Supplementary Movie 7: Localisation of Talin during cell extrusion

Local projection of Talin-GFP in an extruding cell from the midline region of the pupal notum. Scale bar is 5μm.

Supplementary Movie 8: Dynamics of EB1 comets in an extruding cell and its neighbour

Single plane of EB1-GFP in an extruding cell from the midline region of the pupal notum and in the neighbours, fast frame rate (1/sec), concatenated from several movies. Note that the z plane was manually adjusted. Scale bar is 5μm.

Supplementary Movie 9: Depletion of apical MTs in an extruding cell

Local projection of Jupiter-GFP (total tubulin, green, right) and E-cad-tdTomato (magenta, middle) in an extruding cell from the midline region of the pupal notum. Scale bar is 5μm.

Supplementary Movie 10: Depletion of EB1 in an extruding cell

Local projection of EB1-GFP (MT plus end binding, green, right) and E-cad-tdTomato (magenta, middle) in an extruding cell from the midline region of the pupal notum. Scale bar is 5μm.

Supplementary Movie 11: Depletion of human α-tub in an extruding cell

Local projection of an extruding cell expressing human α-tub-mCherry (green, right) and E-cad-GFP (magenta, middle) in the midline region of the pupal notum. Scale bar is 5μm.

Supplementary Movie 12: Evolution of MTs upon cell apical reduction during large scale ablations

Square laser ablation in a pupal notae expressing E-cad-GFP (middle, magenta) and α-tub-mCherry (right, green). Scale bar is 25μm.

Supplementary Movie 13: Caspase activation and MTs depletion

Local projection of an extruding cell expressing α-tub-mCherry (magenta, middle) and GC3AI (green, middle) in the midline region of the pupal notum. Scale bar is 5μm.

Supplementary Movie 14: MT depletion upon optoDronc activation with or without p35

Local projection of sirTubulin (left), E-cad-tdTomato (middle) and GFP (expressed in optoDronc clones, right) upon caspase activation with optoDronc (top) or upon optoDronc activation combined with p35 expression (inhibitor of effector caspases, bottom). Scale bars is 10μm.

Supplementary Movie 15: Cell shape evolution upon local MT recovery

Local projection of EB1-GFP (green, middle) and E-cad-tdTomato (magenta, right) in a colcemid injected pupae in a control region (top) or in a region exposed to repetive pulses of UV (bottom). Note the recovery of EB1 cometes and the increase of cell apical area. Scale bar is 10μm.

Supplementary Movie 16: Cell shape evolution upon partial MT depletion

Local projection of sqh-mKatex3 (MRLC, green) and a clone expressing UAS-α-tub-GFP (magenta) and the clustering system LARIAT. Upon blue light exposure, GFP forms aggregate and cells tend to reduce their apical area. Scale bar is 10μm.

Supplementary Movie 17: Cell extrusion pattern upon MT depletion in clones by Spastin

Live pupal nota expressing E-cad-GFP (green) with *UAS-RFP* clones (magenta, conditional induction) alone (left) or with UAS-Spastin (right). White dots show extruding cells outside the clone, orange dots extrusions inside the clones. Scale bar is 25μm.

Supplementary Movie 18: Cell extrusion pattern in hid-dsRNA clones upon MTs depletion

Live pupal nota expressing E-cad-GFP (green) with *UAS-hid-dsRNA* clones (magenta) injected with water and ethanol (left) or colcemid (right). White dots show extruding cells outside the clone, orange dots extrusions inside the clones. Scale bar is 25μm.

Supplementary Movie 19: Examples of extrusion events with or without caspase activation

Example of extruding cells from *UAS-hid-dsRNA* clones (marked with his-mIFP, 3rd column, greyscale) in a pupae expressing E-cad-tdTomato (magenta, left), the effector caspase sensor UAS-GC3Ai in the clones (green, right), and injected with colcemid. Top: an extrusion without sign of GC3Ai activation, bottom, an extrusion positive for GC3Ai. Scale bar is 10μm.

Supplementary Movie 20: Cell constriction in optoDronc p35 upon colcemid injection

Local projection of E-cad-tdTomato (middle, magenta) and GFP (expressed in optoDronc clones, right, green) upon Caspase9 activation with optoDronc while inhibiting effector caspases with p35. Top movie is a mock-injected pupae, bottom movie is a pupae injected with colcemid. Scale bars is 10μm.

Supplementary Movie 21: Cell extrusion upon DMSO or Taxol+DMSO injection

Local projection of live pupal nota expressing E-cad-3XmKate (magenta, middle) with UAS-EB1-GFP under the control of pnr-gal4 (green, right) injected with DMSO (top) or DMSO + taxol (bottom). Green dots show extruding cells. Scale bar is 25μm.
