# Supplementary material for: Microtubule disassembly by caspases is an important rate-limiting step of cell extrusion
Source: Nat Commun. 2022 Jun 25;13:3632. doi: 10.1038/s41467-022-31266-8 (PMC9233712; doi:10.1038/s41467-022-31266-8)
Supplement: Supplementary file 25 — Reporting Summary [file 41467_2022_31266_MOESM25_ESM.pdf]

nature research

Corresponding author(s): Romain Levayer
Last updated by author(s): May 18, 2022

Reporting Summary

Nature Research wishes to improve the reproducibility of the work that we publish. This form provides structure for consistency and transparency in reporting. For further information on Nature Research policies, see our Editorial Policies and the Editorial Policy Checklist.

Statistics

For all statistical analyses, confirm that the following items are present in the figure legend, table legend, main text, or Methods section.

n/a	Confirmed	
☐	☒	The exact sample size (*n*) for each experimental group/condition, given as a discrete number and unit of measurement
☐	☒	A statement on whether measurements were taken from distinct samples or whether the same sample was measured repeatedly
☐	☒	The statistical test(s) used AND whether they are one- or two-sided<br>*Only common tests should be described solely by name; describe more complex techniques in the Methods section.*
☒	☐	A description of all covariates tested
☐	☒	A description of any assumptions or corrections, such as tests of normality and adjustment for multiple comparisons
☐	☒	A full description of the statistical parameters including central tendency (e.g. means) or other basic estimates (e.g. regression coefficient) AND variation (e.g. standard deviation) or associated estimates of uncertainty (e.g. confidence intervals)
☐	☒	For null hypothesis testing, the test statistic (e.g. *F*, *t*, *r*) with confidence intervals, effect sizes, degrees of freedom and *P* value noted<br>*Give P values as exact values whenever suitable.*
☒	☐	For Bayesian analysis, information on the choice of priors and Markov chain Monte Carlo settings
☒	☐	For hierarchical and complex designs, identification of the appropriate level for tests and full reporting of outcomes
☒	☐	Estimates of effect sizes (e.g. Cohen's *d*, Pearson's *r*), indicating how they were calculated

Our web collection on statistics for biologists contains articles on many of the points above.

Software and code

Policy information about availability of computer code

Data collection	The data were collected and analysed using Matlab R2020a (image analysis and output analysis). The images were collected on a Zeiss confocal LSM880 using the Zen black software (release 14.0.0.0) or a Gataca spinning disc using Metamorph.
Data analysis	Data analysis were performed on Matlab R2020a using built in functions which are clearly described in the methods. The codes used for local projection are already published and quoted in the manuscript (LocalZprojector Fiji plugin, https://gitlab.pasteur.fr/iah-public/localzprojector). The plugins used for segmentation and tracking (on Fiji) are also quoted in the methods, including EPySeg (https://github.com/baigouy/EPySeg) and Tissue analyzer (https://github.com/baigouy/tissue_analyzer). We also used the CorrBleach Fiji macro (https://www.embl.de/eamnet/html/bleach_correction.html) for correcting bleaching when necessary. All the other pipelines used for analysis are available on the following github link which is specified in the Code availability section of the manuscript, and associated with a doi [https://doi.org/10.5281/zenodo.6545302]

For manuscripts utilizing custom algorithms or software that are central to the research but not yet described in published literature, software must be made available to editors and reviewers. We strongly encourage code deposition in a community repository (e.g. GitHub). See the Nature Research guidelines for submitting code & software for further information.

Data

Policy information about availability of data

All manuscripts must include a data availability statement. This statement should provide the following information, where applicable:

- Accession codes, unique identifiers, or web links for publicly available datasets
- A list of figures that have associated raw data
- A description of any restrictions on data availability

All the raw data used for the graphs are available (in a single Source Data excel file, one sub per pannel). The raw movies (local projection) and raw images are deposited on Zotero, associated with a doi and accessible through the following link [https://doi.org/10.5281/zenodo.6546831]

Field-specific reporting

Please select the one below that is the best fit for your research. If you are not sure, read the appropriate sections before making your selection.

☒ Life sciences ☐ Behavioural & social sciences ☐ Ecological, evolutionary & environmental sciences

For a reference copy of the document with all sections, see nature.com/documents/nr-reporting-summary-flat.pdf

Life sciences study design

All studies must disclose on these points even when the disclosure is negative.

Sample size	No specific statistical technic was used to define the sample size. The number of cell quantified was estimated based on the known variability of the extrusion process and the spontaneous fluctuations of cell perimeter and based on our previous experience (Levayer et al. Current Biology 2016, Moreno et al. Current Biology 2019, Valon et al. Dev Cell 2021). We observed higher cell to cell variability rather than a pupae to pupae variability in most of the extruding process and as such averaged data one several pupae without distinction.
Data exclusions	No data was excluded from the analysis (exept when the sample was clearly dead upon injection)
Replication	All the experiments were replicated at least twice and showed reproducible results.
Randomization	We have not used specific randomization technic for sample allocation since we used exclusively isogenic lines. We do not think it is relevant to study covariate in our analysis.
Blinding	We have not sampled or analysed the data blindly as most of the treatment used are associated with very obvious phenotype. All the data were quantified without distinction which should remove any experimentalist bias. Accordingly, we have not used any specific sampling or data exclusions, except in conditions where the pupae was obviously dead (for microinjection).

Reporting for specific materials, systems and methods

We require information from authors about some types of materials, experimental systems and methods used in many studies. Here, indicate whether each material, system or method listed is relevant to your study. If you are not sure if a list item applies to your research, read the appropriate section before selecting a response.

Materials & experimental systems

n/a	Involved in the study	
☐	☒	Antibodies
☐	☒	Eukaryotic cell lines
☒	☐	Palaeontology and archaeology
☐	☒	Animals and other organisms
☒	☐	Human research participants
☒	☐	Clinical data
☒	☐	Dual use research of concern

Methods

n/a	Involved in the study	
☒	☐	ChIP-seq
☒	☐	Flow cytometry
☒	☐	MRI-based neuroimaging

Antibodies

Antibodies used	The following antibody was used: mouse anti acetylated-Tubulin (1/200, Sigma T7451, clone 611B1). Secondary antibody was Life technologies secondary antibodies produced in goat with Alexa 633 (1/100, Life technologies, A21052, lot 1712097), dissected nota were mounted in Vectashield with DAPI (Vectorlab).

Validation	This antibody has been used in several publication in Drosophila (e.g.: Booth et al., Dev Cell 2014, Roper lab) and showed an obvious specificity for microtubules

Eukaryotic cell lines

Policy information about cell lines

Cell line source(s)	S2R+ (DGRC stock 150, RRID:CVCL_Z831) were used in this study
Authentication	The line was not authenticated and was only use to test the tubulin fusion construct with mutations
Mycoplasma contamination	The line was not test for mycoplasma contamination
Commonly misidentified lines (See ICLAC register)	No commonly misidentified lines were used in this study

Animals and other organisms

Policy information about studies involving animals; ARRIVE guidelines recommended for reporting animal research

Laboratory animals	The study used Drosophila melanogaster strains carrying various insertion, mostly starting from the w118 line. All the lines are specified in the table1 of the manuscript and associated with the RRID whenever possible or with the relevant reference. We used without distinction male sand females (as this does not influence extrusion behaviour), we mostly used pupal stage between 16h and 32h post pupal formation.
Wild animals	No wild animals were used in this study
Field-collected samples	The study does not use field-collected sample
Ethics oversight	No need for ethical approvance for Drosophila work

Note that full information on the approval of the study protocol must also be provided in the manuscript.
